# Complete Genome Sequence of Super Biofilm-Elaborating *Staphylococcus aureus* Isolated in Japan

**DOI:** 10.1128/genomeA.01043-17

**Published:** 2017-10-12

**Authors:** Liansheng Yu, Junzo Hisatsune, Hideki Hirakawa, Emiri Mizumachi, Atsushi Toyoda, Koji Yahara, Motoyuki Sugai

**Affiliations:** a Department of Bacteriology, Hiroshima University Graduate School of Biomedical & Health Sciences, Hiroshima City, Hiroshima, Japan; b Project Research Center for Nosocomial Infectious Diseases, Hiroshima University, Hiroshima, Japan; c Kazusa DNA Research Institute, Kazusa-Kamatari, Kisarazu, Chiba, Japan; d Comparative Genomics Laboratory, National Institute of Genetics, Mishima, Shizuoka, Japan; e Department of Bacteriology II, National Institute of Infectious Diseases, Musashimurayama, Tokyo, Japan

## Abstract

*Staphylococcus aureus* JP080, previously named TF2758, is a clinical isolate from an atheroma and a super biofilm-elaborating strain whose biofilm elaboration is dependent solely on polysaccharide poly-*N*-acetylglucosamine/polysaccharide intercellular adhesin (PNAG/PIA). Here, we report the complete genome sequence of strain JP080, which consists of one chromosome and one circular plasmid.

## GENOME ANNOUNCEMENT

*Staphylococcus aureus* is a common human pathogen causing skin and mucosal infections. Colonization of *S. aureus* has been regarded as a risk factor for developing subsequent infections. Some chronic infections, such as endocarditis and osteomyelitis, and those caused by contaminated implanted medical devices, are characterized as biofilm diseases. *S. aureus* biofilm is composed of three major components, the polysaccharide poly-*N*-acetylglucosamine (PNAG), also known as polysaccharide intercellular adhesion (PIA), cell surface and secreted bacterial proteins, and extracellular nucleic acid, which form extracellular polymeric substances (1). We evaluated the biofilm-elaborating ability of clinical isolates in Japan and demonstrated that dependence on these three components for biofilm elaboration varies by strain (2). Strain JP080, previously named TF2758 (3), is an isolate from an atheroma and the highest biofilm producer among the tested isolates, and its biofilm elaboration was dependent solely on PNAG/PIA (2). Upon transcriptional analysis of JP080, we found a novel transcriptional regulator gene, *rob*, with a nonsense mutation in an operon of highly transcribed gene sets in JP080 and reported it as a novel regulator controlling biofilm elaboration (3). In order to elucidate the mechanism underlying the super biofilm elaboration of JP080, whole-genome sequencing was performed.

Genomic DNA of JP080 was extracted using the lysostaphin and QIAamp DNA minikit (Qiagen, Germany) according to the manufacturer’s instructions. DNA libraries were prepared for sequencing with KAPA HyperPlus kits (Nippon Genetics Co., Ltd., Tokyo, Japan). The genome of JP080 was sequenced using a PacBio RS II sequencer and Illumina MiSeq platform. *De novo* assembly using HGAP3 produced two circular contigs composed of a chromosome and a plasmid. To reform sequencing errors, we mapped the Illumina reads to the assembled PacBio contigs. The complete genome sequence was automatically annotated using the Microbial Genome Annotation Pipeline (MiGAP) (4) and manually corrected using *in silico* molecular cloning genomics edition software (In Silico Biology, Inc., Yokohama, Japan) (5).

The JP080 genome is comprised of a 2,729,352-bp chromosome and 37,965-bp plasmid (pJP080), with G+C contents of 33.0% and 30.1%, respectively. The chromosomal genome contained 2,548 predicted protein-coding sequences (CDSs), 60 tRNA genes, and 5 rRNA operons. pJP080 encoded 48 predicted CDSs. The protein sets were functionally annotated using the BLAST program (http://blast.ncbi.nlm.nih.gov).

In the genotyping analysis, JP080 was classified as sequence type 291 (ST291) (by multilocus sequence typing [MLST] analysis), coagulase type VII, and *agr* type I and was negative for *mecA*, β-lactamase, and many other antibiotic resistance genes. The JP080 genome also contained a staphylococcal pathogenicity island, vSaδ, carrying genes encoding exfoliative toxin D (ETD) and epidermal cell differentiation inhibitor B (EDIN-B) (6). pJP080 was a conjugal plasmid coding one putative restriction enzyme and one putative serine protease.

The complete genome sequence of JP080 will contribute to further understanding of the regulatory and signaling pathways of PIA-dependent biofilm formation by *S. aureus*.

### Accession number(s).

The whole-genome sequences of *S. aureus* JP080 and pJP080 have been deposited in DDBJ/EMBL/GenBank under accession no. AP017922 and AP017923 for the chromosome and plasmid, respectively. The versions described in this paper are the first versions.
